# Astragaloside IV Attenuates High-Glucose-Induced Impairment in Diabetic Nephropathy by Increasing Klotho Expression via the NF-*κ*B/NLRP3 Axis

**DOI:** 10.1155/2023/7423661

**Published:** 2023-05-22

**Authors:** Jiaxin He, Jialin Cui, Yimin Shi, Tao Wang, Junyan Xin, Yimeng Li, Xiaomeng Shan, Zhiyao Zhu, Yanbin Gao

**Affiliations:** ^1^Department of Endocrinology, School of Traditional Chinese Medicine, Capital Medical University, Beijing, China; ^2^Department of Endocrinology, Beijing Key Laboratory of Traditional Chinese Medicine Collateral Disease Theory Research, Beijing, China

## Abstract

**Objective:**

Deficiencies in klotho are implicated in various kidney dysfunctions including diabetic nephropathy (DN) related to inflammatory responses. Klotho is closely related to inflammatory responses and is a potential target for ameliorating kidney failure. Pyroptosis, an inflammatory form of programmed cell death, is reported to take part in DN pathogenesis recently. This study is aimed at exploring whether and how klotho inhibited podocyte pyroptosis and whether astragaloside IV (AS-IV) protect podocyte through the regulation of klotho.

**Materials and Methods:**

SD rat model of DN and conditionally immortalized mouse podocytes exposed to high glucose were treated with AS-IV. Biochemical assays and morphological examination, cell viability assay, cell transfection, phalloidin staining, ELISA, LDH release assay, SOD and MDA detection, MMP assay, ROS level detection, flow cytometry analysis, TUNEL staining assay, PI/Hoechst 33342 staining, immunofluorescence assay, and western blot were performed to elucidate podocyte pyroptosis and to observe the renal morphology.

**Results:**

The treatment of AS-IV can improve renal function and protect podocytes exposed to high glucose. Klotho was decreased, and AS-IV increased klotho levels in serum and kidney tissue of DN rats as well as podocytes exposed to high glucose. AS-IV can inhibit DN glomeruli pyroptosis *in vivo*. *In vitro*, overexpressed klotho and treatment with AS-IV inhibited pyroptosis of podocytes cultured in high glucose. Klotho knockdown promoted podocyte pyroptosis, and treatment with AS-IV reversed this effect. Furthermore, the overexpression of klotho and AS-IV reduces oxidative stress levels and inhibited NF-*κ*B activation and NLRP3-mediated podocytes' pyroptosis which was abolished by klotho knockdown. In addition, both the ROS inhibitor NAC and the NF-*κ*B pathway inhibitor PDTC can inhibit NLRP3 inflammasome activation. NLRP3 inhibitor MCC950 can inhibit pyroptosis of podocytes exposed to high glucose.

**Conclusion:**

Altogether, our results demonstrate that the protective effect of AS-IV in upregulating klotho expression in diabetes-induced podocyte injury is associated with the inhibition of NLRP3-mediated pyroptosis via the NF-*κ*B signaling pathway.

## 1. Introduction

Diabetic nephropathy (DN) is a major microvascular complication of diabetes. It is the most common cause of chronic kidney failure and end-stage renal disease and can also increase the risk of cardiovascular disease in diabetic patients. Consequently, DN seriously impacts life expectancy and is a global health burden [1, 2]. Even strict metabolic management and hypertension control cannot completely prevent the progressive decline in renal function [3]. The onset and progression of proteinuria are characteristic of DN and contribute to the decline in renal function [4]. Podocytes, terminally differentiated visceral epithelial cells that maintain the structure and permselectivity function of the glomerular filtration barrier, are vulnerable to injury and have poor regenerative capacity. Their loss and injury are closely related to proteinuria glomerular disease, including DN [5, 6]. Therefore, it is important to study how podocyte damage relates to DN pathogenesis and to explore drugs that effectively promote their regeneration.

Klotho is an antiaging secretory protein with various biological properties such as antiaging and anti-inflammatory effects; it is mainly distributed in the distal convoluted tubules of the kidney [7]. Klotho deficiency is observed in acute and chronic kidney disease including DN [8, 9]. Past studies on klotho mainly focused on the kidney tube; recently, the role of klotho in glomerulopathy has been attracting increasing attention. Moreover, klotho was found to reduce proteinuria in mice with chronic kidney failure and reverse podocyte apoptosis treated with high glucose [10].

Cell inflammation is a key factor in DN pathogenesis. Recently, the strategy of ameliorating disordered inflammation in DN patients has attracted attention [11]. Although many studies have reported klotho's inhibitory effects in inflammatory responses, its effects on DN are unclear. Pyroptosis is an inflammatory form of programmed cell death, with the classical activation pathway relying on caspase-1 activation [12]. The NLR family pyrin domain containing 3 (NLRP3) inflammasome is an important regulatory factor in caspase-1-dependent pyroptosis in DN pathogenesis [13]. Interleukin- (IL-) 18 and IL-1*β* are the primary effectors of pyroptosis; ample evidence suggests their involvement in the development of DN [14]. In type 2 diabetes, saturated fatty acids and high blood sugar activate the NLRP3 inflammasome [15]. More specifically, the overproduction of reactive oxygen species (ROS) induced by high-glucose levels is the main cause of chronic mild inflammation and promotes the activation of NLRP3 inflammatory small bodies [16]. Previous studies on klotho in DN have focused on inhibiting cell apoptosis. In contrast, recent studies have reported that apoptosis and pyroptosis can both damage nephrons in two ways in diabetic mice [17]. Moreover, in nonischemic dilation cardiomyopathy, the rate of pyroptosis in myocardial muscle cells is higher than apoptosis [18]. Although klotho has been reported to improve diabetic cardiomyocyte damage by inhibiting the NLRP3 inflammasome and alleviating diabetic cardiomyocyte fibrosis [19], whether it can improve diabetic podocyte injury by inhibiting pyroptosis remains unclear.

Astragaloside IV (AS-IV) is an active ingredient of Astragalus membranaceus, which has been found to improve cardiovascular disease, liver fibrosis, and DN. Additionally, growing evidence suggests that jaundice methylsine has anti-inflammatory, antioxidative stress, and antiapoptotic effects [20, 21]. Indeed, researchers have previously reported that jaundice methionine has a protective effect on DN mice [22], but the mechanism was not elucidated. AS-IV protected DN rats by inhibiting NF-*κ*B activation and its key downstream inflammatory mediators [23]. On this foundation, we want to explore whether AS-IV inhibited NF-*κ*B by upregulating klotho. Recently, AS-IV has been reported to inhibit NLRP3 inflammatory body-mediated inflammation via TLR4/NF-*κ*B/CaSR to improve vascular endothelial injury [24] and attenuate gestational diabetes mellitus via targeting NLRP3 inflammasome in diabetic mice [25]. However, the mechanism of AS-IV regulating NLRP3 has not been elucidated. Despite the various investigations of AS-IV, we want to further explore whether and how AS-IV inhibits the pyroptosis by inhibiting the NLRP3 inflammasome in podocytes of DN. The foundation of the study is based on klotho; therefore, the relationship between klotho and pyroptosis was emphasized in our paper. Xing et al. have proved that AS-IV can increase levels of klotho mediating oxidative stress via the PPAR*γ*-FoxO1 signaling pathway to inhibit podocyte apoptosis [8]. However, it has not explored the relationship between klotho and pyroptosis. Although apoptosis and inflammatory cell death pathways have extensive crosstalk, it is different from pyroptosis.

Klotho administration was observed to have renoprotective effects [26] in the acute kidney injury model, which suggested that it may be a promising strategy for renal recovery. Though according to the report, AS-IV can upregulate levels of klotho in anesthesia-induced apoptosis in the brain of rats [27] as well as in podocytes treated with high glucose. Past studies have mainly focused on the effects of exogenous klotho such as the recombinant human klotho protein, which has short cycles and action times and therefore is difficult to apply clinically [28]. Thus, the purpose of this study was to explore the effects of AS-IV on endogenous klotho expression in DN as well as the relationship and underlying mechanisms between klotho and DN-induced podocyte damage.

## 2. Materials and Methods

### 2.1. Chemical Reagents

AS-IV (purity ≥ 98%) and anti-klotho antibody (SAB3500604) and anti-gasdermin D (GSDMD) (SAB2108448) were obtained from Sigma-Aldrich (St. Louis, MO, USA). IL-18 (A115) and anti-GSDMD antibody (A20197) were purchased from ABclonal Technology (Wuhan, China). NLRP3 (19771-1-AP) and caspase-1 (22915-1-AP) were obtained from Proteintech (Wuhan, China). Rabbit anti-phospho-nuclear-factor kappa-light-chain-enhancer of activated B cell (NF-*κ*B) p65 antibody (3033) was acquired from Cell Signaling Technology. Antibodies against (NF-*κ*B) p65 (ab16502), acetyl-coenzyme A synthetase 2 (ACS2; ab47092), pro-caspase-1 (ab179515), and IL-1*β* (ab9722) were purchased from Abcam (Cambridge, UK). Nephrin antibody (sc-377246) was obtained from Santa Cruz Biotechnology (Shanghai, China). MCC950 (B7946) and Z-YVAD-FMK (YVAD; A8955) were procured from ApexBio Technology (USA). Pyrrolidinedithiocarbamate ammonium (PDTC; S3633) was acquired from Selleck Chemicals (USA). N-Acetyl-L-cysteine (NAC; S0077) was provided by Beyotime (Shanghai, China).

### 2.2. Animals and Treatments

Eight-week-old male Sprague Dawley rats (180–220 g) were purchased from Weitonglihua (Beijing, China) and reared in individual metabolic cages kept at a constant temperature (24°C) and humidity (70%) and at a controlled 12 h light/dark cycle. All animal experiments conformed to the Guide for the Care and Use of Laboratory Animals from the National Institute of Health, and all procedures in this study were approved by the Institutional Animal Care and Use Committee at the Capital Medical University. After one week of acclimatization, an intraperitoneal injection of streptozotocin (55 mg/kg; Sigma-Aldrich) and a high-fat diet (10% lard, 20% sucrose, 2.5% cholesterol, 0.5% sodium cholate, and 67% basic feed) were used to establish a type 2 diabetes model, as described in a previous study [29]. The control group was injected with the same volume of a sodium citrate buffer and fed a normal diet (12% fat, 28% protein, and 60% carbohydrate). After 7 d, random blood glucose (RGB) levels were assayed using a portable glucometer (Accu-Chek Performa; Roche) in blood obtained from the caudal vein. RGB levels above 16.7 mmol/L for three consecutive days were considered indicative of diabetes. The diabetic rats were continually reared with the high-fat diet to induce DN. The rats were subsequently randomly divided into DN group (*n* = 8) and two AS-IV treatment groups, one gavaged with AS-IV at 40 mg/kg/day (*n* = 8) and the other at 80 mg/kg/day (*n* = 8). The normal control (NC; *n* = 8) and DN groups were gavaged with the same volume of aqua distillate. AS-IV and aqua distillate were administered intragastrically for 12 consecutive weeks.

### 2.3. Biochemical Assays and Morphological Examination

After treatment, the animals were euthanized. The blood samples were collected to measure levels of blood urea nitrogen (BUN), serum creatinine (SCr), glycosylated hemoglobin (HbA1c), total cholesterol (TC), and triglyceride (TG). Timed (24 h) urine was collected for albumin detection. Kidney tissue was fixed with the corresponding fixative solutions for Oil Red O stain and electron microscope analysis. A combination of biochemical analysis and histopathology was used to confirm the establishment of the DN model.

### 2.4. Cell Culture and Treatment

The conditionally immortalized mouse podocyte cell line (BNCC337685) provided by the Bena Culture Collection (Beijing, China) was cultured in Dulbecco's Modified Eagle Medium (Gibco, Carlsbad, CA, USA) with recombinant interferon gamma (PeproTech, London, UK) and 10% fetal bovine serum (Gibco) in a cell incubator set at 33°C for cellular proliferation. Afterwards, the cells were cultured at 37°C with 5% CO_2_ over 7 d for cellular differentiation. When the differentiated podocytes reached 80% confluence, the cells were cultured without serum for 24 h. The cells were divided into the following groups: control (5.6 mmol/L glucose), mannitol (5.6 mmol/L glucose+24.5 mmol/L mannitol), high-concentration glucose (30 mmol/L glucose), AS-IV (AS-IV concentrations at 50, 75, or 100 *μ*mol/L), high glucose+AS-IV (AS-IV concentrations at 50, 75, or 100 *μ*mol/L), high glucose+AS-IV (75 *μ*mol/L), high glucose+pEZ-klotho, high glucose+AS-IV (75 *μ*mol/L)+si-klotho, high glucose+YVAD (20 *μ*mol/L), high glucose+MCC950 (NLRP3 inflammasome inhibitor, 10 ng/mL), high glucose+PDTC (NF-*κ*B inhibitor, 10 *μ*mol/L), and high glucose+NAC (1 mM). AS-IV was dissolved in 0.1% dimethyl sulfoxide and cocultured with the podocytes 12 h prior to high-concentration glucose exposure.

### 2.5. Klotho Overexpression and Small Interfering (si) Ribonucleic Acid (RNA) Transfection

When podocyte fusion reached 60% confluence, they were transiently transfected klotho plasmid EX-Mm34493-M02 (GeneCopoeia, Guangdong, China) or Klotho siRNA (KeyGEN BioTECH, Nanjing, China) using EndoFectin™ MAX transfection reagent (EF003; GeneCopoeia). Following transfection, klotho overexpression and knockdown efficiency in the podocytes were assessed via western blot and quantitative reverse transcription polymerase chain reaction (qRT-PCR). The most efficient knockdown siRNA was chosen for subsequent experiments.

The klotho sequences were as follows: forward: 5′-CAGCCTCCGGACTCTAGC-3′, reverse: 5′-TAATACGACTCACTATAGGG-3′. The siRNA sequences targeting klotho were as follows: mklotho si-1 sense: GCGACUACCCAGAGAGUAUTT; mklotho si-1 antisense: AUACUCUCUGGGUAGUCGCTT; mklotho si-2 sense: GCAGCUUCUGUCUUGGAUATT; and mklotho si-2 antisense: UAUCCAAGACAGAAGCUGCTT.

After transfection, the cultured cells were treated with high glucose and the different reagents for an additional 48 h.

### 2.6. Cell Viability Assay

The podocytes were cultured in 96-well plates for 24 h (5 × 10^3^/well). First, to test AS-IV cytotoxicity, the podocytes were incubated with AS-IV at different doses for 12, 24, 36, or 48 h. Afterwards, the cells were treated with 30 mM glucose with or without AS-IV for 24 or 48 h. Mannitol was used to eliminate the effect of osmosis pressure changes on cells. Then, the cells were incubated with Cell Counting Kit-8 solution (10 *μ*L/well) for a 1 h reaction (Dojindo, Tokyo, Japan). The optical density of each well was measured at 450 nm.

### 2.7. Immunofluorescence and Phalloidin Staining

Podocytes were fixed with 4% cold paraformaldehyde for 30 min. They were then permeabilized with 0.3% Triton X-100 (Dingguo, Beijing, China) for 10 min, followed by blocking with 5% goat serum for 1 h. Then, the cells were reacted with antibodies against nephrin (1 : 50), klotho (1 : 100), GSDMD-N (1 : 200), caspase-1 (1 : 100), and p-NF-*κ*B (1 : 200) and followed by incubation with their corresponding secondary antibodies (1 : 100; ZSGB-bio, Beijing, China) for 2 h at 37°C in darkness. After counterstaining with 4′,6-diaminidino-2-phenylindole (Solarbio, Beijing, China) for 5 min, the sections were imaged using a laser confocal microscope (TCS SP8 STED; Leica, Wetzlar, Germany).

For phalloidin staining, podocytes were incubated with methanol-free formaldehyde at room temperature for 20 min. Then, the fixed cells were permeabilization in 0.1% Triton X-100 for 5 min. After three rinses, the cells were stained with 100 *μ*L/well of phalloidin conjugate working solution (23119; AAT Bioquest, Sunnyvale, CA, USA) at room temperature for 90 min. Subsequently, the cells were sealed and imaged under a laser scanning confocal microscope (TCS SP8 STED; Leica).

### 2.8. Enzyme-Linked Immunosorbent Assay (ELISA)

An ELISA kit (EK4446; SAB, China) was used to measure serum levels of klotho. IL-1*β* (KE10003; Proteintech) and IL-18 levels (ab216165; Abcam) in the podocyte supernatant were quantified using their respective commercial ELISA kits detected via a microplate reader. All operations were performed according to the manufacturer's instructions.

### 2.9. Lactate Dehydrogenase (LDH) Release Assay

LDH concentrations in the cultured podocyte supernatant were measured via an LDH cytotoxicity assay kit (ab197004; Abcam). After the treatments, the supernatant was collected and centrifuged and then was plated in 96 microplates measured at 450 nm using a microplate reader.

### 2.10. Superoxide Dismutase (SOD) and Malondialdehyde (MDA) Detection

SOD and MDA levels in kidney tissue were, respectively, detected via SOD (A001-1) and MDA (A003-1) kits (Nanjing Jiancheng, China).

### 2.11. Mitochondrial Membrane Potential

After the treatments as described above, cells from the different groups were stained with the JC-1 staining solution (C2006; Beyotime) for 20 min at 37°C, followed by two washes with buffer. Then, the cells were analyzed via a FACSCalibur flow cytometer (BD Biosciences). At least three independent experiments were performed.

### 2.12. Measurement of Intracellular ROS Levels

Intracellular total ROS levels in the podocytes were detected using a ROS assay kit (S0033; Beyotime) performed as the manufacturer's protocols. Cells from the different groups in six-well plates were incubated with 10 *μ*mol/L 2′,7′-dichlorodihydrofluorescein diacetate (DCFH-DA) at 37°C for 20 min. Subsequently, ROS levels were quantified using either a flow cytometer (BD Biosciences) or a fluorescence microscope (Leica).

### 2.13. Western Blot Analysis

The total protein from rat renal cortex and cultured podocytes on ice was extracted. The protein samples were separated via sodium dodecyl sulphate–polyacrylamide gel electrophoresis and transferred to polyvinylidene fluoride membranes. The membranes were blocked with 5% skimmed milk on a shaker at room temperature for 1 h followed by overnight incubation at 4°C with the following primary antibodies: anti-procaspase-1 (1 : 1000), anti-caspase-1 (1 : 5000), anti-IL-1*β* (1 : 2000), anti-NF-*κ*B p65 (1 : 1000), anti-nephrin (1 : 1000), anti-IL-18 (1 : 2000), anti-klotho (1 : 1000), anti-NLRP3 (1 : 1000), anti-ACS2 (1 : 2000), anti-GSDMD (1 : 1000), and anti-glyceraldehyde 3-phosphate dehydrogenase (GAPDH; 1 : 20000). The next day, the membranes were incubated with anti-mouse or anti-rabbit horseradish peroxidase-conjugated antibody (1 : 5000; Dingguo, Beijing, China) at room temperature for 2 h. The bands were visualized via an enhanced chemiluminescence kit (Pierce), after which the protein bands were detected using a Quantity One system (Bio-Rad, Hercules, CA).

### 2.14. qRT-PCR

Renal cortex and cultured podocyte total RNA was extracted using TRIzol reagent and reverse transcribed according to the manufacturer's instructions. The qRT-PCR analysis was performed in triplicate. The relative expression levels of klotho and nephrin messenger ribonucleic acid were assessed using the 2−*ΔΔ*Ct method and normalized to GAPDH expression. Real-time polymerase chain reaction primers were designed and synthesized as follows:

R-GAPDH-S: CTGGAGAAACCTGCCAAGTATG

R-GAPDH-A: GGTGGAAGAATGGGAGTTGCT

R-klotho-S: TCCCTCCTTTACCTGAGAACCA

R-klotho-A: CACATCCCACAGATAGACATTCG

R-nephrin(rz)-S: CGTGCTAAAGGCGAGTTCCA

R-nephrin(rz)-A: GGATGAAGGTGATGTCAGGTGC

### 2.15. Flow Cytometry Analysis for Cell Pyroptosis

A FLICA 660 Caspase-1/PI Assay kit (9122; ImmunoChemistry Technologies, USA) was used to detect the pyroptosis rate of podocytes. Cells in six-well plates were collected and were subsequently incubated with FLICA 660 working fluid at 37°C for 1 h in darkness. Following, cells were incubated with a propidium iodide (PI) agent for 10 min. Finally, a flow cytometer (BD Biosciences) was used to detect and analyze the staining.

### 2.16. Terminal Deoxynucleotidyl Transferase dUTP Nick-End Labeling (TUNEL) Staining Assay

A TUNEL detection kit (Roche Diagnostics, Mannheim, Germany) was used to evaluate deoxyribonucleic acid (DNA) fragmentation injury in glomeruli and podocytes cultured in vitro. Briefly, after the kidney sections were deparaffinized, dehydrated, permeabilized, and boiled for antigen retrieval, they were incubated with TUNEL reaction reagents at 37°C as recommended by the manufacturer. After staining, the TUNEL signals were detected and photographed under a microscope.

### 2.17. PI/Hoechst 33342 Staining

After treatment, cells in six-well plates were incubated with Hoechst 33342 solution (KGA212; KeyGEN BioTECH) at 37°C for 10 min. Next, they were washed with staining buffer followed by incubation with PI solution at room temperature for 10 min. After PI incubation, the stained cells were observed and photographed using a fluorescence microscope (Leica).

### 2.18. Statistical Analysis

SPSS v19.0 software (IBM, Armonk, New York, USA) was used for the statistical analysis. Values are presented as mean ± standard deviation of at least three independent experiments. Student's unpaired *t*-tests were used to compare two groups, while a one-way analysis of variance was applied for multiple group comparisons. *P* values < 0.05 were considered to be statistically significant.

## 3. Results

### 3.1. AS-IV Ameliorates Renal Insufficiency in DN Rats

**Figure 1:**
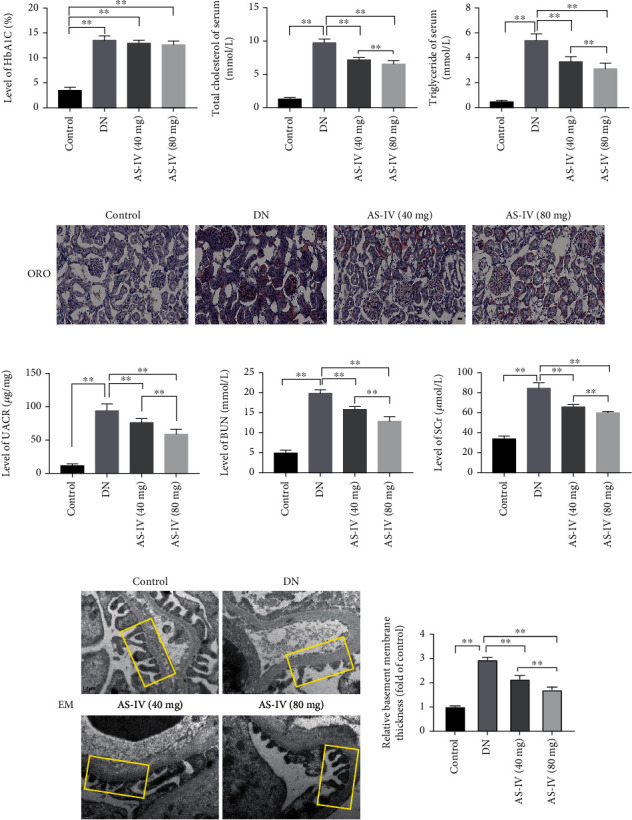
The effect of AS-IV (astragaloside IV) on renal function in DN (diabetic nephropathy) rats. (a–c) The effect of AS-IV on blood sugar and blood lipid levels of DN rats. Scale bar: 25 *μ*m. (d) The effect of AS-IV on UACR (urine albumin creatinine ratio), BUN (blood urea nitrogen), and SCr (serum creatinine), biochemical markers reflecting renal function. (e) The effects of AS-IV on morphology as observed in electron microscopy (8,000x magnification, *n* = 5). GBM (glomerular basement membrane) is depicted in the yellow box. Scale bar: 0.2 *μ*m. Values are presented as mean ± SD (*n* = 8, ^∗∗^*P* < 0.01).

Based on HbA1c levels (Figure 1(a)), AS-IV did not demonstrate a significant hypoglycemic effect in accordance with previous reports [20]. However, as hyperlipidemia is present in type 2 diabetes, we measured serum TG and TC levels of DN rats and used Oil Red O staining to visualize renal lipopathy. As depicted in Figure 1(b), after AS-IV treatment, compared to the control group, TC and TG levels were reduced, and renal lipopathy was dose dependently alleviated (Figure 1(c)). Furthermore, in the AS-IV groups, SCr and BUN levels and the urine albumin creatinine ratio were decreased (Figure 1(d)), suggesting that AS-IV can ameliorate kidney dysfunction. The transmission electron microscopy results revealed that in DN rats, compared to the control group, the glomerular basement membrane in the DN group became significantly thicker and was accompanied by extensive podocyte fusion and even partly disappeared; these effects were reversed by AS-IV treatment (Figure 1(e)). Altogether, the results indicate that AS-IV can improve kidney function. The effect of the AS-IV (80 mg/kg) group is better than that of the AS-IV (40 mg/kg) group.

### 3.2. AS-IV Upregulates Klotho Levels and Protects DN Glomeruli as well as Podocytes Exposed to High Glucose

**Figure 2:**
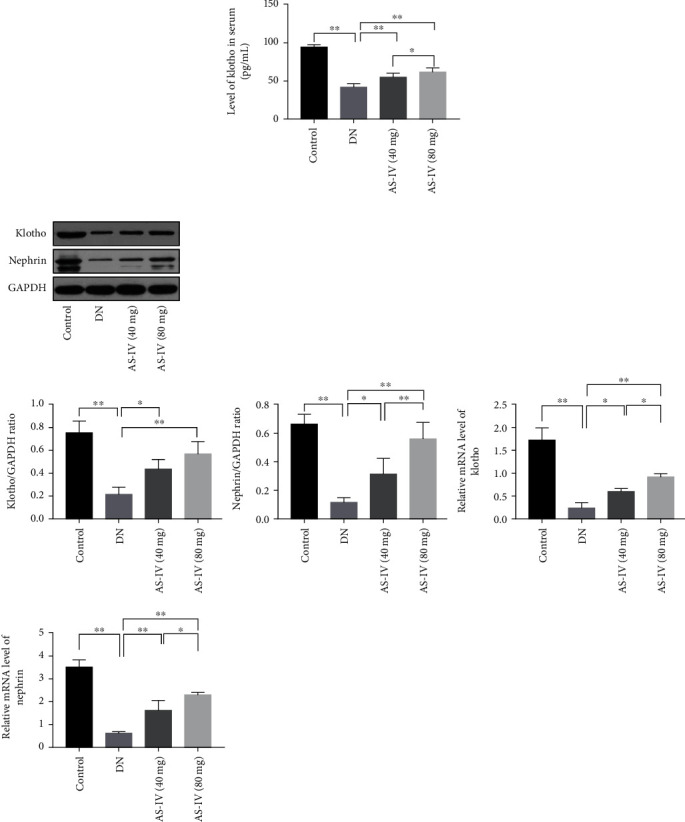
AS-IV upregulates klotho in DN rats. (a) AS-IV upregulates klotho expression in the serum of DN rats. (b) AS-IV treatment enhances klotho and nephrin expression in DN glomeruli. Values are presented as mean ± SD (*n* = 3, ^∗^*P* < 0.05 and ^∗∗^*P* < 0.01).

As evidenced by the ELISA (Figure 2(a)), klotho level in the serum was significantly decreased in DN rats, which is consistent with previous reports [30], and the decrease was reversed by AS-IV. We also conducted western blot and qRT-PCR to detect the expression of klotho and nephrin in kidney glomeruli of DN rats (Figure 2(b)). The results suggest that AS-IV upregulated both klotho and nephrin expression in glomeruli.

**Figure 3:**
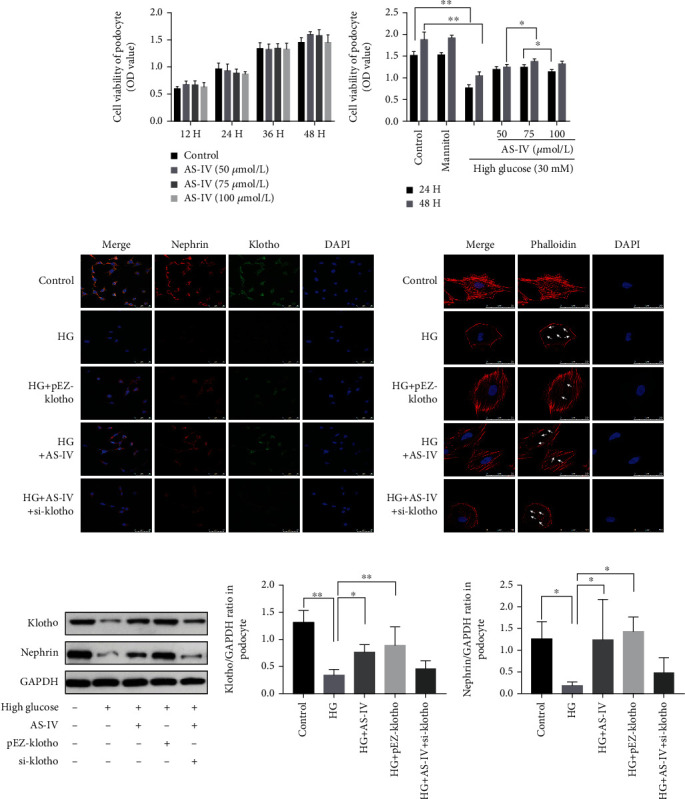
AS-IV upregulates klotho in cultured podocytes exposed to high glucose. (a) The effect of AS-IV on cell viability in podocytes exposed to high glucose. (b) AS-IV treatment increases klotho and nephrin expression in podocytes exposed to high glucose via immunofluorescence staining (630x magnification, *n* = 3). Scale bar: 50 *μ*m. (c) AS-IV treatment ameliorates morphological changes in podocytes exposed to high glucose. Scale bar: 50 *μ*m. (d) AS-IV treatment increases klotho and nephrin expression in podocytes exposed to high glucose via western blot assay. Values are presented as mean ± SD (*n* = 3, ^∗^*P* < 0.05 and ^∗∗^*P* < 0.01).

To further verify our results, we also conducted an in vitro assay. As shown in Figure 3(a), AS-IV showed no obvious drug toxicity and can increased cell viability in podocytes exposed to high glucose. Based on the cell viability results, we chose for subsequent tests an AS-IV concentration of 75 *μ*mol/L and an intervention period of 48 h. Podocytes treated with high glucose expressed lower klotho compared to control podocytes, and this effect was reversed by AS-IV (Figures 3(b) and 3(d)). The results from western blot (Figure 3(d)) and the double immunofluorescence staining (Figure 3(b)) indicated that AS-IV treatment upregulated nephrin and klotho expression in injured podocytes. Furthermore, using cytoskeleton staining (Figure 3(c)), in normal podocytes, the observed cytoskeleton showed a strong network actin stress fiber after upon phalloidin staining. In the model group, high glucose caused reorganization of the actin filaments: dissolution or absent of the cytoplasmic radial stress fibers and redistribution as peripheral bundles to subcortical regions, leading to a polygonal cellular shape. AS-IV was observed to improve skeleton fractures, morphological changes, and cell collapse in podocytes exposed to high glucose. Altogether, this data suggests that AS-IV increases klotho expression and protects podocytes from injury.

### 3.3. AS-IV Protects DN Rat Glomeruli and Podocytes Exposed to High Glucose by Inhibiting Pyroptosis

**Figure 4:**
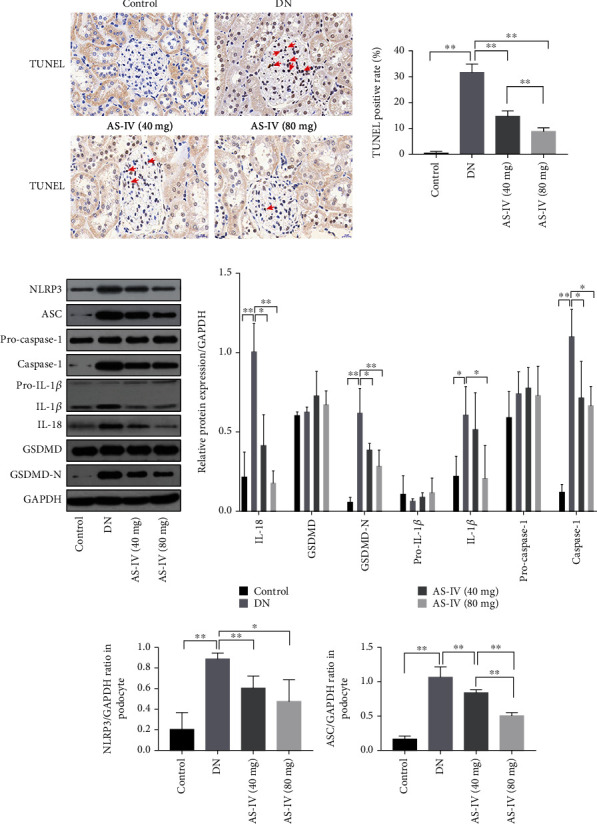
AS-IV inhibits pyroptosis in DN glomeruli. (a) The effect of AS-IV on DNA (deoxyribonucleic acid) fragmentation in DN glomeruli as evidenced by TUNEL (terminal deoxynucleotidyl transferase dUTP nick-end labeling) staining (200x magnification, *n* = 3). Scale bar: 20 *μ*m. (b) The effect of AS-IV on pyroptosis-related parameters in DN kidneys. Values are presented as mean ± SD (*n* = 3, ^∗^*P* < 0.05 and ^∗∗^*P* < 0.01).

To explore whether pyroptosis was associated with the progression of DN glomeruli injury, we assessed DNA fragmentation using TUNEL staining and also quantified the expression levels of proteins related to pyroptosis. As shown in Figure 4(a), the TUNEL staining revealed DNA damage in DN glomeruli, and both AS-IV doses alleviated the injury. As both apoptosis and pyroptosis can cause DNA damage, we further quantified the expression of pyroptosis-related proteins. The western blot results (Figure 4(b)) indicated that IL-1*β* and IL-18 were significantly upregulated in the glomeruli of DN rats compared to healthy control. Furthermore, the results confirmed that caspase-1, the core protein of the classic pyroptosis pathway [31], and GSDMD-N, the primary effector protein in pyroptosis [32], were both upregulated in DN glomeruli compared to control rats (Figure 4(b)). These data suggests that pyroptosis is occurring in the DN glomeruli and that AS-IV intervention can dose dependently reverse it.

**Figure 5:**
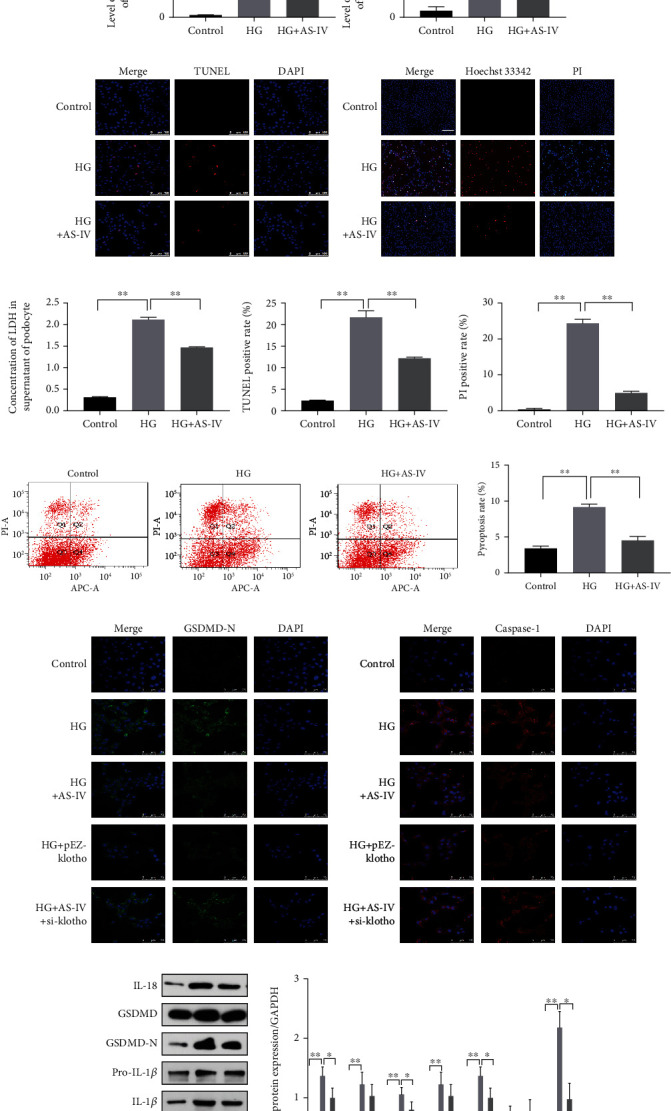
AS-IV inhibits pyroptosis in podocytes treated with high glucose. (a) Effect of YVAD (Z-YVAD-FMK) on cell survival in podocytes exposed to high glucose. (b) The effect of AS-IV on expression of IL-1*β* and IL-18 in podocyte supernatant. (c) TUNEL staining to detect DNA fragmentation in podocytes in vitro (200x magnification, *n* = 3). Scale bar: 100 *μ*m. (d) PI (propidium iodide)/Hoechst 33342 staining (200x magnification, *n* = 3). Scale bar: 50 *μ*m. (e) The effect of AS-IV on LDH (lactate dehydrogenase) release in podocyte supernatants. (f) Caspase-1/PI staining as assayed by flow cytometry. (g) GSDMD-N and caspase-1 immunofluorescence (630x magnification, *n* = 3). Scale bar: 75 *μ*m. (h) The effect of AS-IV on pyroptosis-related parameters in podocytes exposed to high glucose. Values are presented as mean ± SD (*n* = 3, ^∗^*P* < 0.05 and ^∗∗^*P* < 0.01).

To confirm that pyroptosis is associated with high-glucose-induced podocyte injury, YVAD, a caspase-1 specific inhibitor, was added to podocytes exposed to high glucose. We found that YVAD can significantly increase cell survival rate (Figure 5(a)), suggesting that pyroptosis is involved in high-glucose-induced podocyte injury. We used PI/Hoechst 2334 staining and a LDH release assay to detect pore formation and cell content release while concurrently performing TUNEL staining to observe DNA fragmentation. As shown in Figure 5(b), AS-IV inhibits IL-1*β* and IL-18 release, improved DNA fragmentation (Figure 5(c)), inhibited pore formation, and decreased LDH release in podocytes treated with high glucose (Figures 5(d) and 5(e)). Moreover, AS-IV significantly downregulated the caspase-1/PI positive rate (Figure 5(f)). We also quantified the related pyroptosis index, as illustrated in Figure 5(h). The results indicate that compared to the control group, high glucose induced caspase-1, IL-1*β*, IL-18, and GSDMD-N terminal upregulation in podocytes, and after AS-IV treatment, the expression of the protein was downregulated. The caspase-1 and GSDMD-N immunofluorescence staining (Figure 5(g)) were consistent with the in vivo results, further confirming the results of our experiment.

### 3.4. NLRP3-Mediated Pyroptosis in Podocytes Exposed to High Glucose

**Figure 6:**
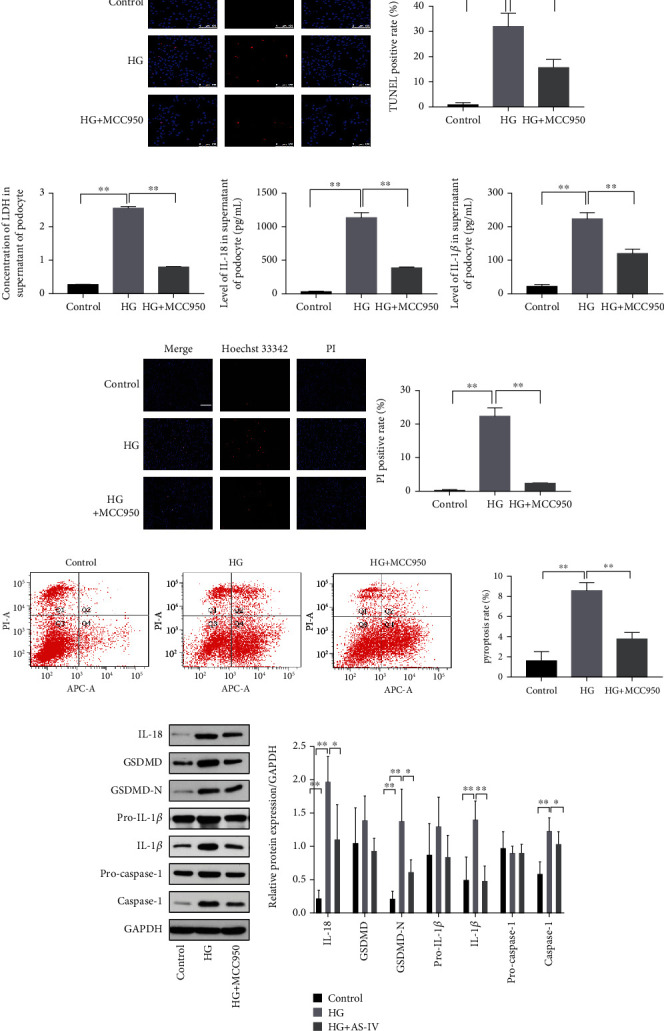
NLRP3 (NLR family pyrin domain containing 3) inhibitor blocks pyroptosis in high-glucose-treated podocytes. (a) The effect of MCC950 on TUNEL staining (200x magnification, *n* = 3). Scale bar: 100 *μ*m. (b) The effect of MCC950 on LDH release in podocyte supernatant. (c) The effect of MCC950 on the expression of IL-1*β* and IL-18 in podocyte supernatants. (d) The effect of MCC950 on PI/Hoechst 33342 staining (200x magnification, *n* = 3). Scale bar: 50 *μ*m. (e) The effect of MCC950 on caspase-1/PI staining as assayed using flow cytometry. (f) The effect of MCC950 on pyroptosis-related proteins in podocytes exposed to high glucose. Values are presented as mean ± SD (*n* = 3, ^∗^*P* < 0.05 and ^∗∗^*P* < 0.01).

The activation of the NLRP3 inflammasome is closely related to pyroptosis. Thus, to confirm whether the pyroptosis observed in the podocytes is associated with NLRP3 inflammasome activation, we cocultured cells with a specific NLRP3 inhibitor, MCC950. Moreover, TUNEL staining revealed that MCC950 inhibited DNA fragmentation in injured podocytes (Figure 6(a)) and decreased the expression of IL-1*β* and IL-18 in podocyte supernatant (Figure 6(c)). Using PI/Hoechst 33342 staining and an LDH release assay, we found that MCC950 inhibited pore formation and attenuated LDH release in podocytes exposed to high glucose (Figures 6(d) and 6(b)). As shown in Figure 6(f), MCC950 application downregulated the expression of pyroptosis-related proteins and caspase-1/PI staining (Figure 6(e)) positive rate in podocytes treated with high glucose. Accordingly, the above data demonstrates that NLRP3 inhibition can inactivate caspase-1-mediated pyroptosis, suggesting that NLRP3 inflammasome activation is involved in the pyroptosis of podocytes exposed to high glucose.

### 3.5. AS-IV Can Upregulate Klotho to Reduce Oxidative Stress Levels and NF-*κ*B Activation in High-Glucose-Treated Podocytes and Can Alleviate Oxidative Stress Levels and NF-*κ*B Activation in DN Glomeruli

**Figure 7:**
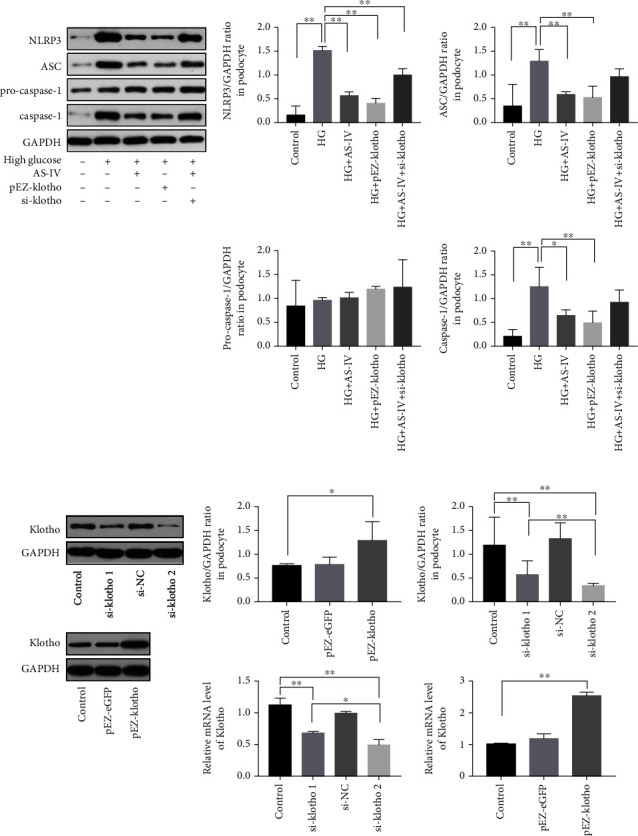
Effect of AS-IV on NLRP3 inflammasome activation in vitro. (a) The effect of AS-IV on NLRP3 inflammasome activation in vitro. (b) The mRNA level and the protein level by qRT-PCR and western blot assay. Values are presented as mean ± SD (*n* = 3, ^∗^*P* < 0.05 and ^∗∗^*P* < 0.01).

Based on the results above, we speculated that the effects of AS-IV on DN are associated with klotho upregulation. To elucidate its function, we used plasmids to transfect podocytes with klotho so that they overexpress it. The transfection efficiency was verified using western blot and qRT-PCR (Figure 7(b)). As Figure 3(c) illustrates, klotho improved cytoskeletal reorganization in podocytes exposed to high glucose. Additionally, klotho upregulation increased nephrin protein levels (Figures 3(b) and 3(d)) and protected podocytes from injury.

**Figure 8:**
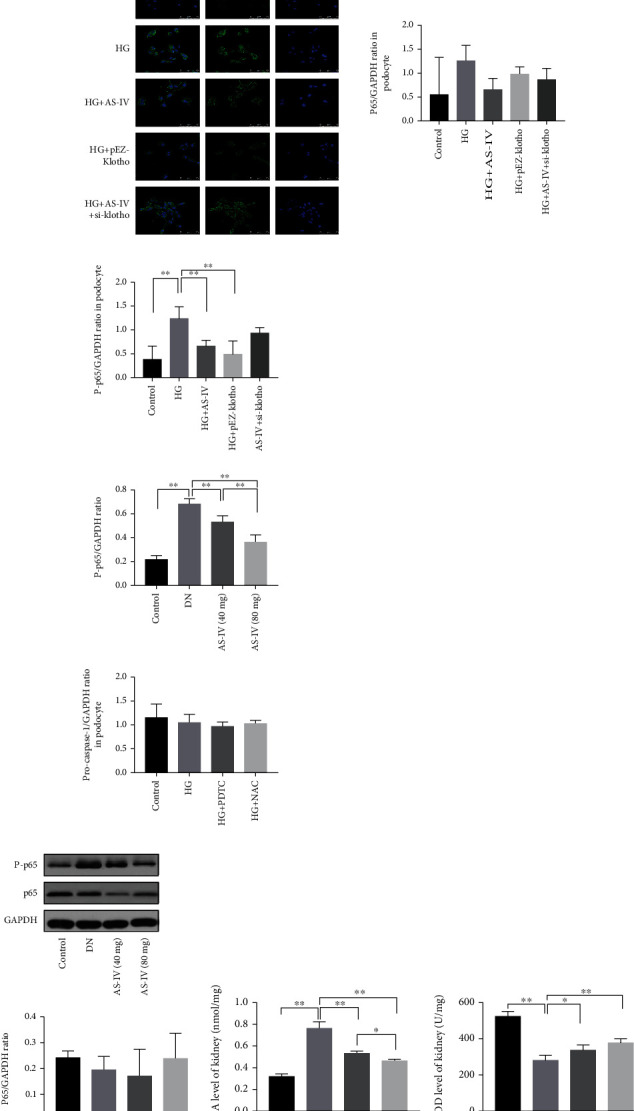
AS-IV alleviates oxidative stress levels and NF-*κ*B (nuclear-factor kappa-light-chain-enhancer of activated B cells) activation in podocytes exposed to high glucose and in DN glomeruli. (a) ROS (reactive oxygen species) was quantified via DCFH-DA (2′,7′-dichlorodihydrofluorescein diacetate) staining coupled with flow cytometry or immunofluorescence (200x magnification, *n* = 3). Scale bar: 50 *μ*m. (b) The mitochondrial membrane potential as assayed via flow cytometry. (c, d) NF-*κ*B activation in podocytes was detected using immunofluorescence and western blot (630x magnification, *n* = 3). Scale bar: 75 *μ*m. (e) NF-*κ*B activation in DN glomeruli was assessed via western blot. (f, g) MDA (malondialdehyde) and SOD (superoxide dismutase) levels in DN glomeruli as measured via ELISA (enzyme-linked immunosorbent assay). (h) PDTC (pyrrolidinedithiocarbamate ammonium) inhibits NLRP3 inflammasome activation in vitro. Values are presented as mean ± SD (*n* = 3, ^∗^*P* < 0.05 and ^∗∗^*P* < 0.01).

Furthermore, klotho upregulation inhibited ROS accumulation in podocytes (Figure 8(a)) and raised the mitochondrial membrane potential (Figure 8(b)) in high-glucose conditions. Pretreatment with ROS scavenger NAC can reverse the activation of NLRP3 inflammasome complex in Figure 8(h), suggesting that ROS plays an important role in the activation of NLRP3 inflammasome. When AS-IV was applied after klotho siRNA transfection, ROS accumulation in podocytes was increased and the mitochondrial membrane potential was higher compared to AS-IV treatment alone (Figures 8(a) and 8(b)). Similarly, klotho knockout weakened the effect of AS-IV on cytoskeleton reorganization and nephrin expression (Figures 3(c) and 3(d)). Klotho knockout also weakened the effect of AS-IV on NF-*κ*B p65 inhibition (Figures 8(c) and 8(d)) in podocytes treated with high glucose. Altogether, AS-IV, by at least partially increasing klotho expression, alleviated oxidative stress and inhibited NF-*κ*B activation, thereby protecting podocytes exposed to high glucose.

The antioxidant ability of AS-IV in the glomeruli of DN rats was assessed via SOD and MDA content analysis. As depicted in Figures 8(f) and 8(g), compared to the DN group, after AS-IV intervention, MDA content was significantly decreased and SOD activity was significantly higher than in the DN rats. The AS-IV 80 mg/kg group had a stronger effect than the AS-IV 40 mg/kg group. Consistent with the in vitro experiment, we found that AS-IV can also significantly inhibit NF-*κ*B activation in DN glomeruli, as Figure 8(e) illustrates.

### 3.6. AS-IV Upregulates Klotho to Inhibit NLRP3 Inflammasome Activation in Podocytes Treated with High Glucose and Inhibit NLRP3 Inflammasome Activation in DN Glomeruli

As shown in Figure 4(b), AS-IV downregulated the activated NLRP3 inflammasome protein and decreased the expression of caspase-1, NLRP3, and apoptosis-associated speck-like protein (ASC). Given that AS-IV can inhibit NLRP3 inflammasome activation in DN glomeruli and according to previous reports, klotho can improve diabetic cardiomyopathy by inhibiting the NLRP3 inflammasome pathway [19]. We wondered if klotho mediates NLRP3 inflammasome activation in podocytes exposed to high glucose. We measured NLRP3 inflammasome protein levels in vitro, and as shown in Figure 7(a), AS-IV attenuated the upregulation of the NLRP3 inflammasome after high-glucose treatment. Following klotho protein overexpression, the NLRP3 inflammasome complex was significantly downregulated, suggesting that klotho can mediate NLRP3 inflammasome activation. Notably, AS-IV can also downregulate NLRP3 inflammasome expression in vitro, and this inhibition was greatly weakened after klotho protein knockout, implying that AS-IV partly inhibits the NLRP3 inflammasome complex in cultured podocytes via the klotho protein.

### 3.7. AS-IV Inhibits Pyroptosis in High-Glucose-Treated Podocytes via the Klotho-NF-*κ*B-NLRP3 Axis

We further studied the mechanism of AS-IV inhibition of pyroptosis in podocytes exposed to high-glucose environment. We found that PDTC (pyrrolidinedithiocarbamate ammonium) (a NF-*κ*B inhibitor) application significantly inhibited NLRP3 inflammasome activation compared to control cells (Figure 8(h)), suggesting that NF-*κ*B activation is involved in NLRP3 inflammasome activation and ultimately induce podocyte pyroptosis. Furthermore, klotho can mediate NF-*κ*B phosphorylation, indicating that klotho inhibits the NLRP3 inflammasome via blocking NF-*κ*B activation in podocytes exposed to high glucose. Thus, AS-IV may inhibit NLRP3 inflammasome activation via partially blocking klotho-mediated NF-*κ*B activation.

To verify that AS-IV can inhibit pyroptosis by regulating klotho, we reduced klotho protein expression using si-klotho knockdown. Consequently, expression of NLRP3 inflammasome complex in vitro was inhibited via AS-IV intervention, but the inhibition of the NLRP3 inflammasome complex was greatly weakened after klotho protein knockout (Figure 7(a)). Altogether, the results indicate that AS-IV may attenuate podocyte pyroptosis in high-glucose environments via the klotho-NF-*κ*B-NLRP3 axis.

## 4. Discussion

Past studies have demonstrated that strict monitoring of blood glucose and blood lipid levels still cannot completely prevent the progressive decline of renal function in DN patients; this decline may be related to the persistent inflammation caused by the high-glucose and high-fat environment [2]. Based on our results, we speculate that AS-IV improves renal function and protects podocytes independently from glucose mediation; rather, it likely inhibits the inflammation induced by metabolic abnormalities.

Various proinflammatory pathways and inflammatory molecules have been demonstrated to correlate with proteinuria, renal fibrosis, and renal function decline [33], suggesting that metabolic disorders induce chronic low-grade inflammation, which is crucial in DN progression. DN is a chronic inflammatory disease closely associated with podocyte abnormalities. Podocyte loss and damage, a cause of proteinuria in kidney damage, is an important pathological mechanism of DN. Previous studies have focused on podocyte apoptosis, and we also previously reported that regulating podocyte apoptosis can improve kidney function and reduce proteinuria in diabetic mice [34].

The relationship between the pathogenesis of diabetic complications and pyroptosis has recently attracted attention [14]. Pyroptosis, a type of programmed cell death characterized by cytoplasmic destruction, membrane pore formation, DNA fragmentation, and the release of the proinflammatory cytokines IL-1*β* and IL-18, is involved in various acute and chronic kidney diseases, including kidney injury in diabetes mellitus [35]. In this study, we detected pyroptosis, which can also cause podocyte damage and loss in addition to apoptosis; we observed DNA fragmentation in DN glomeruli and in podocytes treated with high glucose, which was accompanied by the formation of cytoplasmic pores and the increased release of the inflammatory cytokines IL-18 and IL-1*β*. These results indicate that pyroptosis was occurring in DN glomeruli and in podocytes cultured in vitro; the pyroptosis was reversed by AS-IV. To further verify that pyroptosis is involved in podocyte injury, we applied pyroptosis inhibitors to podocytes treated with high glucose. Here, the podocytes treated with pyroptosis-specific caspase-1 inhibitors had significantly increased cell survival rates, indicating that pyroptosis is involved in podocyte damage in DN.

Pyroptosis can be initiated nonclassically and classically, which pathway is mediated by an inflammatory body assembly dependent on caspase-1 activation. Among them, the NLRP3 inflammatory complex has been studied comprehensively. The NLRP3 inflammasome, a multiprotein complex, is involved in various renal diseases [12]. It consists of a sensor molecule, NLRP3, an adaptor molecule, ASC, and an effector protein, caspase-1. After activation of the NLRP3 inflammasome, caspase-1 is cleaved, which is followed by the cleavage of the effector protein GSDMD [36]. Accumulating evidence indicates that NLRP3 is involved in diabetes mellitus kidney damage. Notably, NLRP3 inflammasome activation has been detected in endothelial cells, mesangial cells, and podocytes in renal tissues [37, 38]. Leng et al. suggested that AS-IV could inhibit NLRP3 inflammasome activation via inhibiting TLR4/NF-*κ*B signaling pathway and CaSR in STZ-induced diabetic rats and human umbilical vein endothelial cells cultured in high glucose [24]. NLRP3 inflammasome can induce apoptosis as well as pyroptosis. Previous studies focused on apoptosis, but few studies on pyroptosis. The focus of this study is to explore the intervention effect of AS-IV on podocyte pyroptosis. In this study, we found that the NLRP3 inflammasome complex was activated in DN kidney tissue and in podocytes treated with high glucose. To verify whether the NLRP3 inflammasome participates in podocyte pyroptosis in a high-glucose environment, we tested the effect of MCC950, a NLRP3 inhibitor, in vitro. MCC950 inhibited the expression of GSDMD-N, IL-1*β*, and IL-18, implying that high-glucose-induced pyroptosis depends partly on NLRP3 inflammasome activation. GSDMD is recognized as the executor of pyroptosis. It is cleaved into an active N-terminal and an inactive C-terminal following caspase-1-mediated activation of the classical pathway or the activation of the nonclassical pathway mediated by caspase-4, caspase-8, and caspase-11 [39]. The active N-terminal forms a pore in the cytoplasm to promote the release of inflammatory molecules, which amplify the inflammatory reaction and tissue injury [17]. In this study, we found that the expression of cleaved GSDMD-N was increased, and AS-IV can inhibit GSDMD-N expression both in vitro and in vivo.

Klotho was first discovered as an antiaging protein mainly expressed in renal tubular cells [40]. Recently, it was also found in glomeruli, specifically in endothelial cells and podocytes [41]. The overexpression of proinflammatory and inflammatory factors is positively correlated with decreased klotho expression in chronic kidney disease and kidney injury [42]. Following klotho knockout, the TNF-induced expression of adhesion molecules and NF-*κ*B activation increased in endothelial cells in vitro and chronic inflammatory conditions exacerbated [43]. Prior studies have reported that klotho expression is significantly reduced in renal tissues and in the serum of DN patients as well as animal model [44], which was replicated in this study. Additionally, we found decreased klotho levels in DN rat renal and in high-glucose cultured podocytes, while AS-IV significantly increased klotho expression in vivo and in vitro. We speculate that AS-IV improves kidney function in DN rats and protects podocytes, and these effects are closely related to klotho protein upregulation following AS-IV treatment.

Klotho is expressed in multiple cells and shows renal protective properties. Klotho is mainly expressed in renal tubular epithelial cells. Klotho deficiency has been observed in renal tubules of patients and animal models with renal injury. Recently, researchers have found that klotho overexpression or exogenous supplement can alleviate renal fibrosis by suppressing epithelial-to-mesenchymal transition in cultured human renal tubular epithelial cells [45]. Research has shown that the decrease of klotho expression is implicated in the process of diabetes nephropathy by promoting M1 polarization. Overexpression of klotho can regulate macrophages, which is involved in the process of inflammatory response in acute and chronic renal injury, specifically by stimulating macrophage polarization towards the anti-inflammatory M2 phenotype to inhibit the inflammatory response in macrophages and show reno-protective effects [46, 47]. Klotho has been reported to protect the function and viability of endothelial cells, including glomerular endothelial cells injured by high glucose [48]. As is known, the endothelial cell is an important component of vascular cells. Klotho deficiency is implicated in the process of vascular dysfunction or structural abnormalities in the arterial wall, which is closely related to the incidence rate and mortality of chronic kidney disease [49]. According to relevant research, serum and urine klotho levels may be a marker for predicting podocyte damage in human renal tissue [50]. Klotho plays an important role in maintaining the structure, morphology, and function of podocytes. Researches have showed that klotho reduced ATP-stimulated actin cytoskeletal remodeling and transepithelial albumin leakage in podocyte [9] and inhibits apoptosis in HG-treated podocytes [51], which is critical for proper glomerular filtration. Klotho has been shown to mediate the activation of the NLRP3 inflammasome in several diseases. Zhao et al. reported that klotho protein overexpression inhibited the NLRP3/caspase-1 pathway to improve cognitive impairment via the promotion of microglia transformation in an Alzheimer's model [7]. Zhu et al. found that klotho expression is closely related to brain immunity, and klotho protein can inhibit the activation of NLRP3 inflammatory bodies in epithelial cells [52], whereas whether klotho has an effect on the NLRP3 inflammasome in DN and its underlying mechanism is unknown.

Numerous studies have reported that klotho inhibits NF-*κ*B activation. Jin et al. found that klotho attenuates renal inflammation by inhibiting p-p65 expression during nephropathy [28]. Similarly, Jin et al. demonstrated that exogenous klotho can inhibit p65 and NLRP3 activation and reduce proinflammatory cytokine secretion [53]. In this study, NF-*κ*B was significantly inhibited after klotho overexpression, and both klotho overexpression and NF-*κ*B inhibition blocked the expression of NLRP3 inflammasome complex. These results indicate that podocyte pyroptosis as regulated by the klotho/NF-*κ*B signaling pathway plays an important role in DN podocyte damage. In the DN rat model and in high-glucose cultured podocytes, the expression of phosphorylated NF-*κ*B was significantly increased. The application of the NF-*κ*B inhibitor PDTC significantly reduced the expression of NLRP3 inflammasome complex proteins, indicating that podocyte pyroptosis may be regulated by NF-*κ*B. Mitochondrial dysfunction can activate NLRP3 [54]; recently, klotho has been reported to maintain mitochondrial membrane potential stability and inhibit the ROS/thioredoxin-interactin protein/NLRP3 signaling pathway in cardiomyocyte [19]. Depolarization and damage of mitochondrial induced by high glucose are confirmed by the decrease of mitochondrial membrane potential in podocytes in this study. ROS mainly comes from mitochondria; pretreatment with ROS scavenger NAC can reverse the activation of NLRP3 inflammasome complex in our study. These results suggest that ROS plays an important role in the activation of NLRP3 inflammasome in DN podocyte injury. AS-IV and klotho can significantly inhibited production of ROS; however, this effect was attenuated by klotho protein knockdown significantly, suggesting that AS-IV may inhibit ROS production partly through klotho. High glucose increased oxidative stress responses and attenuated klotho expression. Imbalances in oxidant and antioxidant factors and ROS accumulation from mitochondrial dysfunction can activate NF-*κ*B, resulting in NLRP3-activated caspase-1-mediated podocyte pyroptosis. In this study, we also confirmed the inhibitory effect of AS-IV on NF-*κ*B signaling pathway. In addition, we found that AS-IV inhibited NF-*κ*B by upregulating klotho, and NF-*κ*B activation participated in NLRP3-mediated pyroptosis of podocytes cultured in high glucose.

AS-IV is a natural plant component composed of cycloartane-type triterpene glycosides. It is a bioactive saponin mainly extracted from membranaceus roots. AS-IV possesses various biological properties; it has antioxidant and antifibrotic effects, and it also confers immune protection. For example, AS-IV was found to protect BEAS-2B cells from intermittent hypoxia injury via inhibiting proinflammatory cytokine overproduction [55]. We have previously confirmed the renoprotective effect of AS-IV. Nevertheless, its underlying mechanisms in protecting podocytes from DN injury via the inhibition of inflammatory responses remain unclear. Podocyte pyroptosis in DN pathogenesis and progression may be related with decreased klotho and is likely a key mechanism of kidney injury pathogenesis. Few studies exist on the effects of active ingredients from currently known natural medicines on klotho, with some studies reporting on the effects of identified ginseng active ingredients [56] and AS-IV. We revealed here that the renoprotective properties of AS-IV are closely related to klotho upregulation and its antipyroptosis properties.

In conclusion, our results indicate that klotho protein can alleviate high-glucose-induced podocyte injury via inhibiting NLRP3 inflammasome-mediated pyroptosis. AS-IV can protect DN glomeruli and podocytes in a high-glucose environment by upregulating the expression of endogenous klotho protein. The protective effects of AS-IV on podocytes cultured in vitro are mainly related to oxidative stress and NF-*κ*B/NLRP3-induced pyroptosis as regulated by klotho. Therefore, our paper provides a theoretical and pharmacological basis for the renoprotective properties of AS-IV upregulating klotho.

## Data Availability

The data used to support the findings of this study are available from the corresponding author upon request.

## Supplementary Materials

Supplementary Materials The original images of WB files were shown in the Supplementary Materials. Figure S1: AS-IV treatment enhances klotho and nephrin expression in DN glomeruli. Figure S2: AS-IV treatment increases klotho and nephrin expression in podocytes exposed to high glucose via western blot assay. Figure S3: the effect of AS-IV on pyroptosis-related parameters in DN glomeruli. Figure S4: the effect of AS-IV on pyroptosis-related parameters in podocytes exposed to high glucose. Figure S5: the effect of MCC950 on pyroptosis-related proteins in podocytes exposed to high glucose. Figure S6: (a) NF-*κ*B activation in podocytes was detected using western blot; (b) NF-*κ*B activation in DN glomeruli was assessed via western blot; (c) PDTC inhibits NLRP3 inflammasome activation in vitro. Figure S7: the effect of AS-IV on NLRP3 inflammasome activation in vitro. Figure S8: the protein level of klotho was detected using western blot assay.
