# Supplementary material for: Astragaloside IV Attenuates High-Glucose-Induced Impairment in Diabetic Nephropathy by Increasing Klotho Expression via the NF-κB/NLRP3 Axis
Source: J Diabetes Res. 2023 May 22;2023:7423661. doi: 10.1155/2023/7423661 (PMC10228232; doi:10.1155/2023/7423661)
Supplement: Supplementary Materials — The original images of WB files were shown in the Supplementary Materials. Figure S1: AS-IV treatment enhances klotho and nephrin expression in DN glomeruli. Figure S2: AS-IV treatment increases klotho and nephrin expression in podocytes exposed to high glucose via western blot assay. Figure S3: the effect of AS-IV on pyroptosis-related parameters in DN glomeruli. Figure S4: the effect of AS-IV on pyroptosis-related parameters in podocytes exposed to high glucose. Figure S5: the effect of MCC950 on pyroptosis-related proteins in podocytes exposed to high glucose. Figure S6: (a) NF-κB activation in podocytes was detected using western blot; (b) NF-κB activation in DN glomeruli was assessed via western blot; (c) PDTC inhibits NLRP3 inflammasome activation in vitro. Figure S7: the effect of AS-IV on NLRP3 inflammasome activation in vitro. Figure S8: the protein level of klotho was detected using western blot assay. [file 7423661.f1.docx]

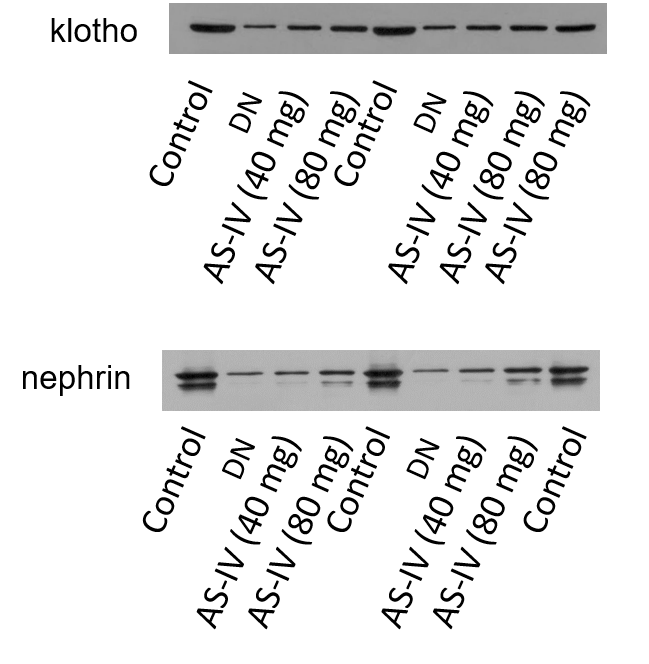


Figure S1. AS-IV treatment enhances klotho and nephrin expression in DN glomeruli.


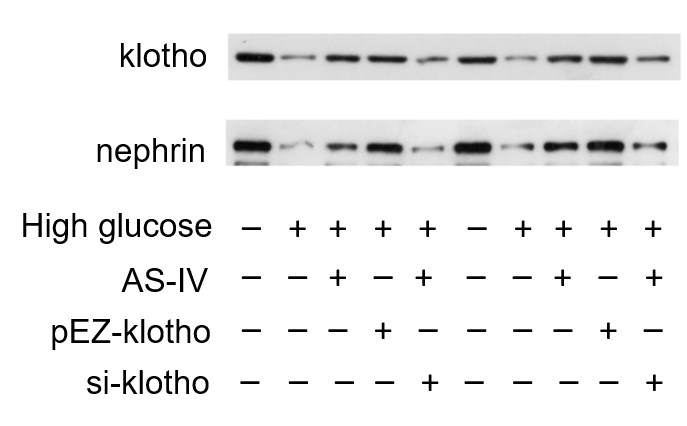


Figure S2. AS-IV treatment increases klotho and nephrin expression in podocytes exposed to high glucose via western blot assay.


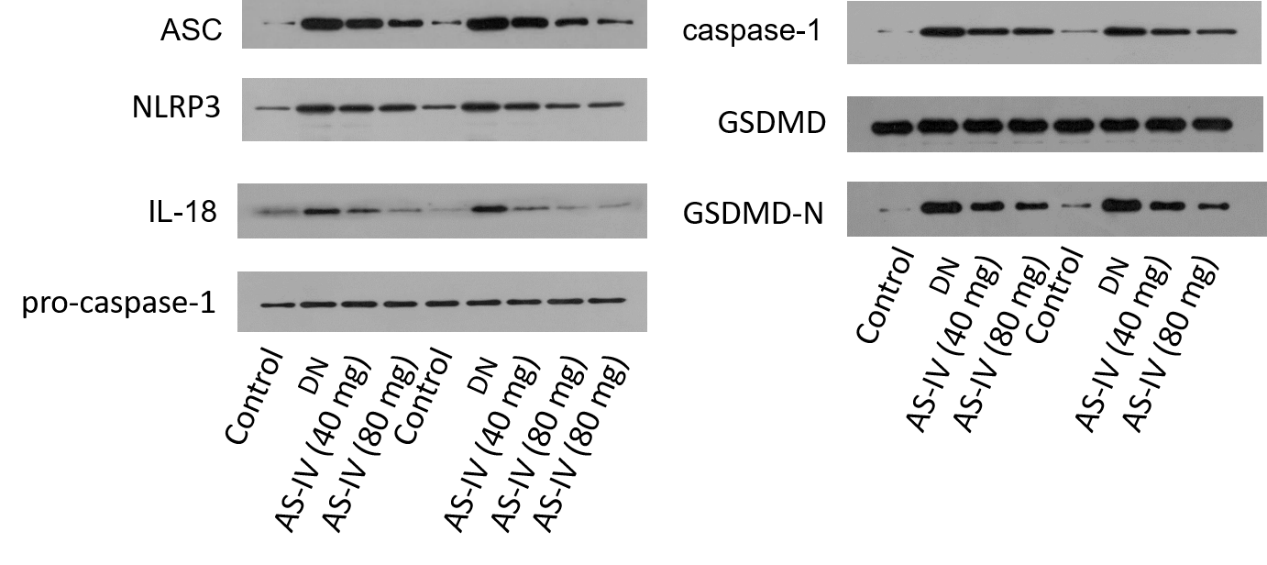


Figure S3. The effect of AS-IV on pyroptosis-related parameters in DN glomeruli.


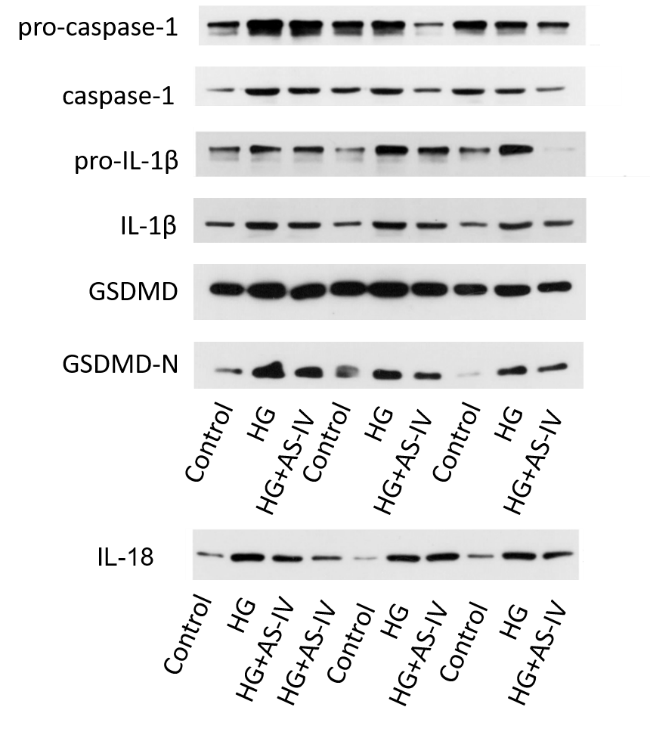


Figure S4. The effect of AS-IV on pyroptosis-related parameters in podocytes exposed to high glucose.


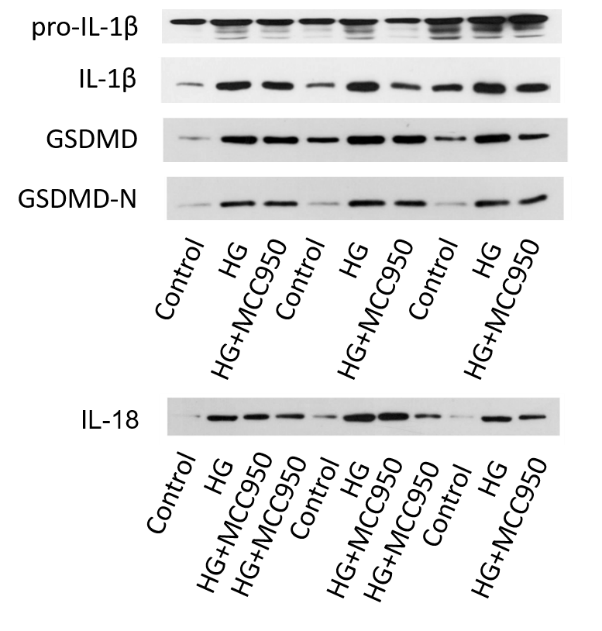


Figure S5. The effect of MCC950 on pyroptosis-related proteins in podocytes exposed to high glucose.


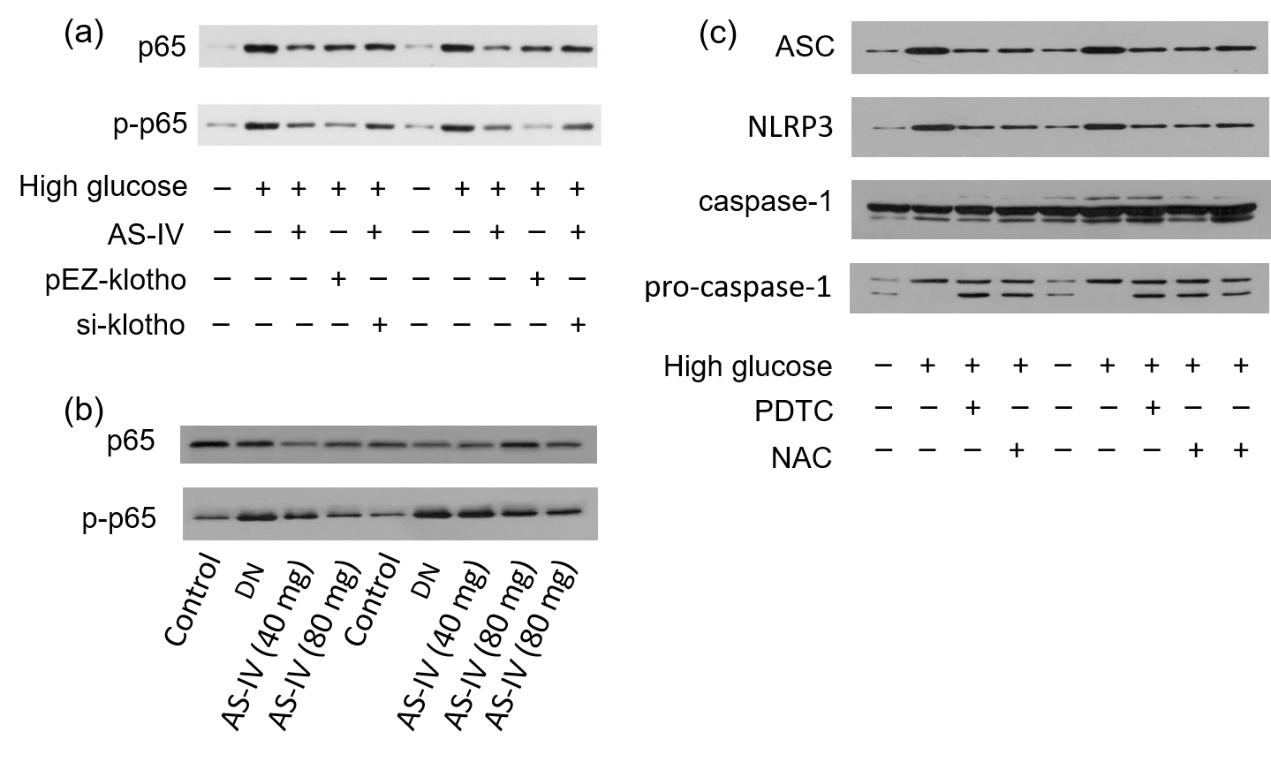


Figure S6. (a) NF-κB activation in podocytes was detected using western blot; (b) NF-κB activation in DN glomeruli was assessed via Western blot; (c) PDTC inhibits NLRP3 inflammasome activation in vitro.


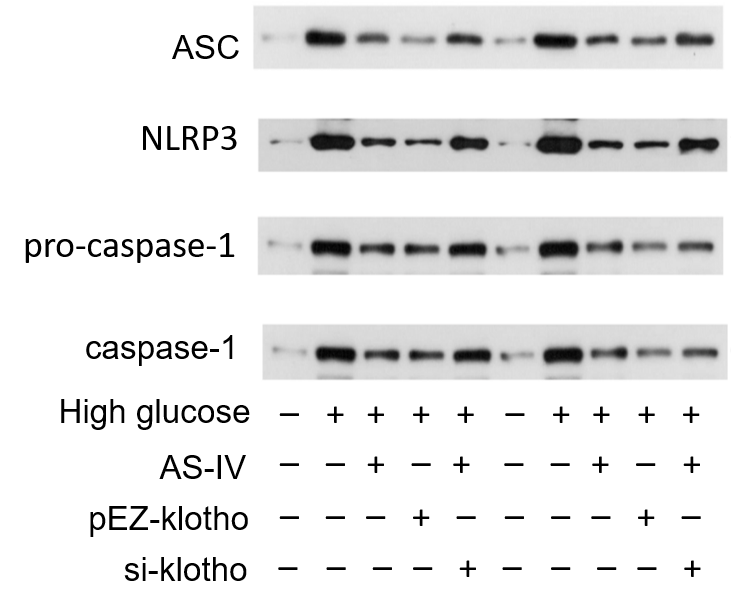


Figure S7. The effect of AS-IV on NLRP3 inflammasome activation in vitro.


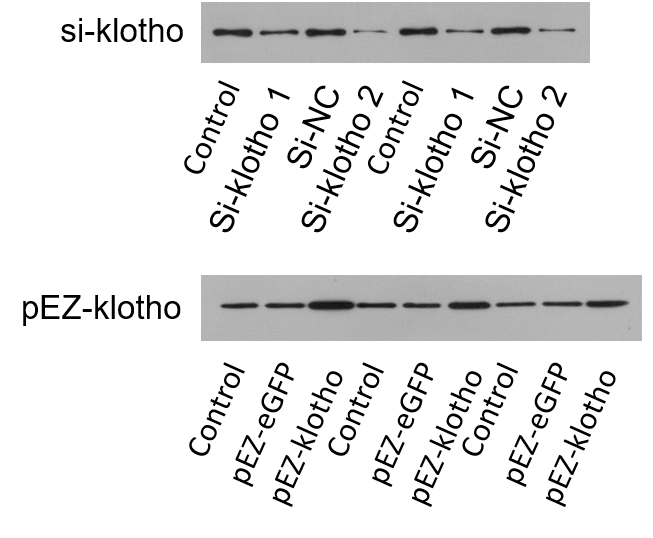


Figure S8. The protein level of klotho was detected using western blot assay.
